# Stillness, Light, and Distance

**DOI:** 10.3201/eid2302.AC2302

**Published:** 2017-02

**Authors:** Byron Breedlove

**Affiliations:** Centers for Disease Control and Prevention, Atlanta, Georgia, USA

**Keywords:** art science connection, emerging infectious diseases, art and medicine, about the cover, fungi, coccidioidomycosis, infectious diseases, shiprock mesa, Maynard Dixon, public health, Valley fever, stillness, light, and distance

**Figure:**
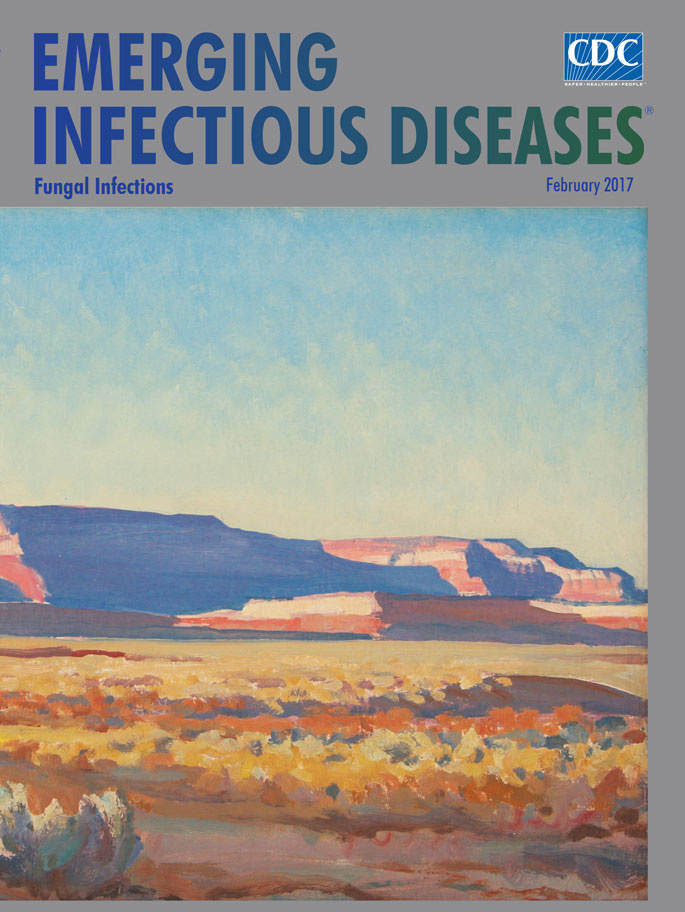
**Maynard Dixon (1875–1946), Shiprock Mesa (1942). Oil on canvas, 12 in × 16 in/30.5 cm × 40.6 cm.** Mark Sublette Medicine Man Gallery, 6872 E. Sunrise Dr, Ste 130, Tucson, AZ, USA.

Maynard Dixon was born near Fresno, California, in the center of the San Joaquin Valley, on January 24, 1875. He had asthma as a child and spent much of his time drawing. In 1891, when he was 16, Dixon sent two of his sketchbooks to Frederic Remington, an artist he admired. Remington encouraged him, replying, “You draw better at your age than I did at the same age. If you have the ‘sand’ to overcome difficulties, you could be an artist in time. No one’s opinion of what you can do is of any consequence—time and your character will develop that.”

Dixon lived and worked for several years in San Francisco. However, according to biographer Donald J. Hagerty, Dixon “periodically roamed the West's plains, mesas, and deserts on foot, horseback, buckboard—even by automobile—drawing, painting, and writing, pursuing a transcendent awareness of the region's spirit.” When Dixon visited Arizona near the beginning of the 20th century, he proclaimed that “he had found his country” and developed his signature style of depicting western themes.

Although the 1906 San Francisco earthquake and fire destroyed his studio and most of his early creations, over his lifetime Dixon created an abundant body of work including illustrations for novels, paintings in several genres, murals, and poetry. His paintings of western US landscapes found throughout are considered remarkable for their realism and detail. Through his art, Dixon also documented economic and social hardships during the Great Depression, lifestyles of Native American cultures, and lingering vestiges of cowboy life. Though Dixon often featured the expansive, remote vistas of the western United States, he typically created these scenes, including “Shiprock Mesa” this month’s cover art, on small canvases.

The mesa dominates this painting, its characteristic flat top and stair-stepped edges etched against a pale, shimmering blue sky. Red and sand-colored strata vibrantly contrast with the immense dark blue shadows playing across the steep cliff faces. The softer shapes and textures of the scrub vegetation that carpets the sandy plains contrast with the bold depiction of the mesa and cloudless sky. Hagerty wrote, “Through long and sympathetic observation, Dixon learned how plains rise and fall as they flow toward the horizon, and how the architecture of mesa and butte marches rhythmically over the landscape into the infinite freedom of a deep blue sky.” 

Dixon’s nearly photographic painting, a relaxing study in stillness, light, and distance, appears deceptively simple. Los Angeles art critic Arthur Millier wrote that “Dixon is so steeped in desert forms and colors that these little pictures appear to come from his brush like effortless lyrics.” In describing Dixon’s landscapes, the Pasadena Museum of California Art notes that “His modernist approach to painting Western landscapes featured simple compositions and powerful color fields that shifted the genre away from the more typically sentimental treatment of familiar subject matter.”

In 1894, three years after Dixon wrote to Remington and a year before he landed his first paying job illustrating western scenes for a newspaper, a case of disseminated coccidioidomycosis, or Valley fever as it is commonly known, was first reported in California. This reemerging infectious disease is caused by *Coccidioides immitis,* a soil fungus native to San Joaquin Valley, Dixon’s birthplace, and by *C. posadasii*, which is common to other arid-to-semiarid areas of the southwestern United States, northern portions of Mexico, and areas of Central America and South America. Approximately 60% of reported cases of coccidioidomycosis in the United States occur in Arizona, Dixon’s beloved “country.”

Coccidioidomycosis is usually acquired by breathing microscopic *Coccidioides* fungal spores released after humans, animals, or the weather disturbs contaminated soil or dust. There has been a striking increase in cases of coccidioidomycosis during the past several decades, including a major outbreak in California in 1991–1994. Some of this increase may be related to greater recognition and diagnosis, as well as improved reporting. Some factors linked to this increase throughout the western expanses, where Dixon traveled and worked, are the upsurge in susceptible human populations including older individuals and those with immune deficiencies from non–coccidioidomycosis-endemic areas; rise of cities and towns in areas with *Coccidiodes*-contaminated soil; occupational exposures; and changing weather patterns.
